# Comparing Confirmed Sodium Nitrite Suicide Deaths With Poison Center Surveillance Estimates

**DOI:** 10.1001/jamanetworkopen.2024.34192

**Published:** 2024-09-23

**Authors:** Joshua Bloom, Alexis Sharpe, Seth Nulman, Katrina Monday, Jeanna M. Marraffa, Catherine Stayton, Carla Clark, Michele Slone, Gail Cooper, Mark K. Su

**Affiliations:** 1 Department of Emergency Medicine, The Warren Alpert Medical School of Brown University, Providence, Rhode Island; 2 Ronald O Perelman Department of Emergency Medicine, NYU-Grossman School of Medicine, New York, New York; 3 NYC Poison Center, NYC Department of Health and Mental Hygiene, New York, New York; 4 NYCH+H/Jacobi + North Central Bronx/Albert Einstein College of Medicine, Bronx, New York; 5 Onondaga County Medical Examiner’s Office, Wallie Howard Jr. Center for Forensic Sciences, Syracuse, New York; 6 Upstate Medical University, Upstate NY Poison Center, Syracuse, New York; 7 Injury and Violence Prevention Program, Bureau of Environmental Health and Injury Prevention, Division of Environmental Health, NYC Department of Health and Mental Hygiene, New York, New York; 8 NYC Office of Chief Medical Examiner, New York, New York

## Abstract

**Importance:**

Sodium nitrite is a curing agent increasingly used for self-harm and suicide, and multiple locales have reported increasing cases. However, approaches to forensic investigation of these cases are not standardized, and current modes of surveillance in the United States may be inadequate.

**Objective:**

To define a potential standard approach for identifying sodium nitrite deaths based on forensic confirmation, and compare findings based on this standard to poison center surveillance.

**Design, Setting, and Participants:**

This retrospective case series of sodium nitrite exposures and deaths was conducted in 2 urban medical examiner jurisdictions in New York State from 2000 to 2022. The population-based sample included individuals from (1) medical examiner reports of decedents where the cause of death was sodium nitrite and (2) poison center reports of intentional exposures to sodium nitrite.

**Exposure:**

Sodium nitrite as either cause of death (medical examiner reports) or intentional exposure (poison center reports).

**Main Outcomes and Measures:**

Medical examiner determination of sodium nitrite deaths was considered the criterion standard and relied largely on confirmatory blood nitrite testing. Poison center records were assessed for intentional exposures to sodium nitrite.

**Results:**

In this case series of 36 decendents, median (range) age was 28 (20-57) years; 23 (63.8%) were male; 6 (16.7%) were African-American, 5 (13.9%) were Chinese, 13 (36.1%) were White, and 4 (11.1%) had unknown race; and 6 (16.7%) were Hispanic. No deaths were found from 2000 to 2018, and yearly increases in deaths from 2019 to 2022; these deaths were largely missed by local poison center surveillance. Most cases (83.3% [n = 30]) had postmortem blood nitrite concentrations available, and multiple decedents had evidence of suicide kit recommendations from internet sources.

**Conclusions and Relevance:**

In this case series of decedents in 2 New York medical examiner jurisdictions, sodium nitrite deaths increased yearly, and the medical examiners were able to obtain confirmatory nitrite concentrations in most cases. These findings suggest that poison center surveillance underestimates confirmed deaths from sodium nitrite; public health authorities should rely on multiple data sources when analyzing this problem, and forensic analyses should be standardized.

## Key Points

**Question:**

Are surveillance estimates of sodium nitrite suicide deaths consistent with cases confirmed by medical examiners?

**Findings:**

In this case series, 36 confirmed sodium nitrite deaths in 2 medical examiner jurisdictions were observed; cases increased from 2019 to 2022, and there were none from 2000 to 2018. These deaths exceed estimates from poison center data, and many decedents had evidence of items commonly recommended as part of a suicide kit.

**Meaning:**

These results suggest that multidisciplinary strategies and collaboration are needed to standardize reporting and surveillance of sodium nitrite deaths.

## Teaser

This case series study defines a new approach for identifying sodium nitrite deaths based on forensic confirmation and compares its findings with those of current poison center surveillance.

## Introduction

Sodium nitrite is a strong oxidizer used in preserving meat, and it is generally available without a license or prescription. Ingesting as little as 1 gram can cause life-threatening methemoglobinemia.^1^ Pro-suicide forums frequently advise coingestion of sodium nitrite with antiemetic drugs and antihistamines to facilitate absorption and to mitigate symptoms that occur after ingestion. Together with a measuring scale, these items can be assembled into a “suicide kit.”^2,3^ Several reports have described an increased trend in intentional sodium nitrite exposures in the United States based on surveillance reporting, with evidence that individuals learn how to use sodium nitrite from the internet.^4,5,6^ Multiple case series have also described clusters of these cases, although methods of confirmation of nitrite as the cause of death are inconsistent.^1,7,8,9,10,11^ This phenomenon is also under investigation by the lay press and public health authorities.^12^ We gathered a case series of sodium nitrite deaths in 2 medical examiner jurisdictions in New York State to investigate the trends and characteristics of these deaths and compare with poison center surveillance.

## Methods

This retrospective case series was approved by the institutional review boards of the NYC Department of Health and Mental Hygiene and Upstate Medical University. Informed consent was waived due to the retrospective nature of the study. This report followed the reporting guideline for acceptable reporting of uncontrolled case series.^13^

**Figure 1.**
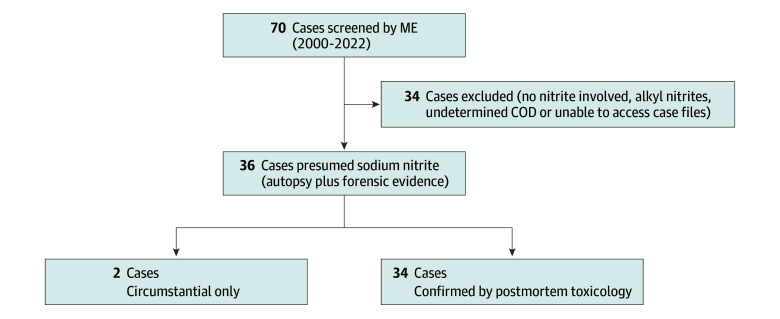
Criteria for Identifying Sodium Nitrite Deaths in UVIS-CMS and BEAST BEAST indicates Bar Coded Evidence Analysis Statistical Tracking; COD, cause of death; ME, medical examiner; UVIS-CMS, Unified Victim Identification-Case Management System.

This case series was based on confirmed sodium nitrite deaths identified by the medical examiners of New York City (NYC) and Onondaga County in New York State. Searches of the Unified Victim Identification-Case Management System (UVIS-CMS) of the Office of Chief Medical Examiner (OCME) in NYC and Bar Coded Evidence Analysis Statistical Tracking (BEAST) in Onondaga County were undertaken using the search terms “nitrite,” “nitrate,” and “methemoglobin,” identifying 70 cases between 2000 and 2022. Thirty-four were excluded as detailed in Figure 1. Our medical examiners prefer blood or tissue nitrite confirmation when assigning this cause of death, but postmortem examination or methemoglobin levels were used when nitrite testing was unavailable. When nitrite concentrations were obtained, a colorimetric assay was performed (NMS Labs) and results greater than 1000 micromoles per liter were considered consistent with nitrite poisoning, although further quantitation was not available. Medical and/or clinical toxicologists (J.B. and J.M.) and forensic toxicologists (G.C. and K.M.) independently reviewed and confirmed these findings, and there was complete agreement.

We also queried the databases of both poison centers in New York State for the same periods and locales where deaths were observed in UVIS-CMS and BEAST. We identified cases with exposure to substances within AAPCC Code 034260 (Nitrates and Nitrites, Excluding Medications and Substances of Abuse), substances containing the plain text “nitrite,” “nitrate,” or “methemoglobin,” and cases that coded methemoglobinemia, therapeutic use of methylene blue, or death as an outcome. Cases without intentional exposure to sodium nitrite or located outside of jurisdictions surveyed in UVIS and BEAST were excluded.

Case data were obtained by manual abstraction of full individual case notes from both sources. Demographic variables such as race and ethnicity were recorded verbatim from death certificates, and sociodemographic information was abstracted for the purpose of identifying and addressing disparities and promoting equity in public health research.

### Statistical Analysis

Descriptive statistics were used in this study to characterize the data. The analysis was conducted between August 2023 and August 2024 using Excel version 16 (Microsoft) and GraphPad Prism version 10 (GraphPad Software).

## Results

Our case series identified 36 sodium-nitrite-related suicide cases in NYC and Onondaga medical examiner (ME) jurisdictions, all from 2019 onward; median (range) age was 28 (20-57) years; 23 (63.8%) were male; 6 (16.7%) were African-American, 1 (2.8%) was Asian Indian, 5 (13.9%) were Chinese, 3 (8.3%) were Filipino, 1 (2.8%) was Irish, 2 (5.6%) were Korean, 1 (2.8%) was Nepalese, 13 (36.1%) were White, and 4 (11.1%) had unknown race; 6 (16.7%) were Hispanic. Thirty-four of these were deemed sodium nitrite deaths based on postmortem confirmation of toxic nitrite or methemoglobin concentrations, and 2 were included based on circumstantial evidence (Figure 1). Only 3 cases were from Onondaga ME jurisdictions: 2 in 2019 and 1 in 2021. Of 520 screened poison center cases, 7 cases were coded as intentional sodium nitrite exposures.

**Figure 2.**
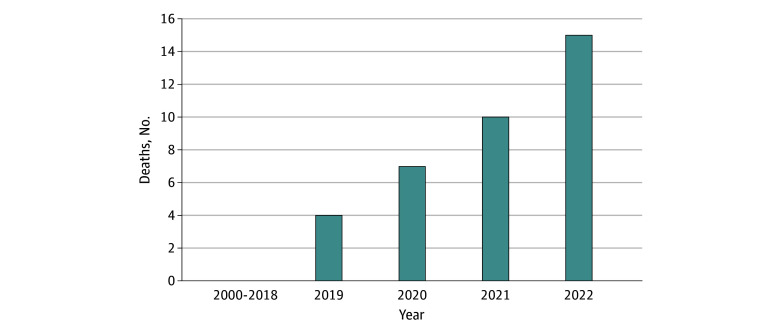
Sodium Nitrite Deaths in New York City and Onondaga Medical Examiner Jurisdictions, 2000-2022

We noted a yearly increase in deaths documented by the MEs from 2019 to 2022 (Figure 2). Of the 7 cases of intentional sodium nitrite exposure reported to local poison centers during this period, only 2 had a major or lethal outcome. The characteristics of sodium nitrite decedents are detailed in the Table. Most cases were found deceased and not taken to a hospital. Of the 5 taken to a health care facility, 1 was reported to a poison center as a sodium nitrite overdose, 1 was reported as a quetiapine overdose, and 3 had no corresponding poison center reports found.

**Table.** Characteristics of Sodium Nitrite Decedents in New York City and Onondaga ME Jurisdictions, 2000-2022

Characteristic	Decedents, No. (%) (N = 36)
Age, median (range), y	28 (20-57)
Sex	
Male	23 (63.8)
Female	11 (30.6)
Unknown	2 (5.6)
Race and ethnicity	
African American	6 (16.7)
Asian Indian	1 (2.8)
Chinese	5 (13.9)
Filipino	3 (8.3)
Irish	1 (2.8)
Korean	2 (5.6)
Nepalese	1 (2.8)
White	13 (36.1)
Unknown	4 (11.1)
Ethnicity	
Hispanic	6 (16.7)
Non-Hispanic	30 (83.3)
Medical profession^a^	5 (13.9)
Found deceased	31 (86.1)
Jurisdiction	
NYC OCME	33 (91.7)
Onondaga ME	3 (8.3)
Postmortem findings	
Nitrite concentration	30 (83.3)
Methemoglobin	6 (16.7)
No internal exam	12 (33.3)
Brown lividity	5 (13.9)
Silver/gray lividity	26 (72.2)
Purple lividity	10 (27.8)
Pink skeletal muscle	1 (2.8)
Chocolate brown blood	23 (63.9)
Forensic findings	
Labeled sodium nitrite product	22 (61.1)
Measuring scale	9 (25.0)
Antiemetic (any)^b^	17 (47.2)
Metoclopramide^c^	6 (16.7)
Antihistamine (any)^d^	12 (33.3)
Cimetidine	3 (8.3)
Evidence of suicide research^e^	6 (16.7)

Abbreviations: ME, medical examiner; OCME, Office of Chief Medical Examiner.

^a^ Includes physician, dentist, nurse, assistant, and technician in health care–related fields.

^b^ Includes antipsychotic drugs, first generation antihistamines, ginger, and standard antiemetic drugs.

^c^ Bottle or pills present at scene, or detected in postmortem toxicology.

^d^ Includes H1 and H2 antagonists.

^e^ Based on forensic investigation and includes websites or the *Peaceful Pill Handbook*.

Twenty-two of 36 (61.1%) of the ME decedent cases had evidence of purchased sodium nitrite found by forensic investigators. Seventeen decedents (47.2%) had an antiemetic found at the scene, and 6 of these were metoclopramide; 12 (33.3%) of the decedents had an antihistamine at the scene, and 3 of these were cimetidine. A measuring scale was present at the scene in 9 (25.0%) of the decedents. Six (16.7%) of the decedents had evidence of suicide research, which included web browsers open to the SanctionedSuicide, other pro-suicide websites, or a copy of *The Peaceful Pill Handbook* (a pro-suicide text).

## Discussion

We found that sodium nitrite–related suicide deaths in New York are increasing. Although national poison data estimated 13 to 20 deaths from 2019 to 2020, our findings confirmed 11 in our local jurisdictions alone during that period.^4,5^ Similarly, a recent National Poison Data System analysis found only 33 deaths out of 124 cases from 2019 to 2022, which is less than those observed in our jurisdictions over the same period.^14^

Local poison centers did not capture these deaths, even when the patients presented to a hospital: only 1 of 5 hospital decedents had a corresponding poison center report. Any putatively reliable method of harm by poisoning would be expected to commonly cause death prehospital; this fact, in addition to poison center reliance on voluntary reporting, may explain our findings. A 2011 report found that only 2.4% of ME-determined poisoning-related fatalities in New York City could be found in the corresponding poison center database^15^; in our case series, only 1 of 36 (2.8%) had a corresponding poison center report identifying sodium nitrite as the exposure.

The National Violent Death Reporting System (NVDRS) has also been used to estimate the national burden of intentional sodium nitrite deaths, finding 260 incidents from 2018 to 2020.^6^ However, NVDRS data are abstracted independently prior to analysis, and multiple states (including New York) were excluded from that analysis due to varying participation in reporting. Additionally, confirmation of these deaths via measurement of nitrite in body fluid or tissue is not standardized among medical examiners.^11^ Our methodology allowed us to directly examine ME and poison center reports to estimate the prevalence in our jurisdictions, which both emphasize nitrite confirmatory testing to determine the cause of death.

At the time of this writing, national legislative efforts aim to generate accountability for hosting of pro-suicide conversations on the internet or create restrictions on the sale of sodium nitrite. The importance of lethal means restriction cannot be underestimated. Cyanide, a poison of similar potency to sodium nitrite, carries a scheduled status in New York State and is not available for general purchase; intentional cyanide-related deaths in our jurisdictions are extremely uncommon. In addition to legislative measures, health care clinicians and public health authorities should maintain awareness of the toxic syndrome and preparedness for its treatment.

### Limitations

This study is subject to multiple limitations. First, there is a risk of coding and categorization errors in a retrospective analysis. Second, there was a lack of postmortem toxicology confirmation in 2 decedents, and 4 early (2019) cases relied on postmortem methemoglobin as opposed to nitrite concentrations (a known source of error in forensics).^1^ Third, poison center surveillance is subject to multiple sources of uncertainty due to errors, omissions, and inconsistency in data reporting, and it is possible that observation in a different locale or timeframe would produce different surveillance results. However, it is encouraging that our data accord with prior investigations of poison center capture of fatalities.^15^

## Conclusions

In this case series of decedents in 2 New York medical examiner jurisdictions, we found a yearly increase in confirmed sodium nitrite deaths that was not reflected in corresponding poison center surveillance. Public health authorities should utilize multiple sources of surveillance to monitor sodium nitrite deaths. Forensic investigators should standardize their evaluation of these deaths, including obtaining postmortem nitrite confirmation when possible. Clinicians should be educated on diagnosing and treating methemoglobinemia, and mental health clinicians should be aware of this agent when evaluating suicide risk.

## Notes

Supplement.
Data Sharing Statement
