# Supplementary material for: Comparing Confirmed Sodium Nitrite Suicide Deaths With Poison Center Surveillance Estimates
Source: JAMA Netw Open. 2024 Sep 23;7(9):e2434192. doi: 10.1001/jamanetworkopen.2024.34192 (PMC11420693; doi:10.1001/jamanetworkopen.2024.34192)
Supplement: Supplement. — Data Sharing Statement [file jamanetwopen-e2434192-s001.pdf]

Data Sharing Statement

Bloom. Comparing Confirmed Sodium Nitrite Suicide Deaths With Poison Center Surveillance Estimates. *JAMA Netw Open*. Published September 23, 2024.
doi:10.1001/jamanetworkopen.2024.34192

Data
Data available: No

Additional Information
Explanation for why data not available: PHI was analyzed during this study and will not be made publicly available. Deidentified or aggregate data can be made available on reasonable request.
